# Dissection of a DNA-damage-induced transcriptional network using a combination of microarrays, RNA interference and computational promoter analysis

**DOI:** 10.1186/gb-2005-6-5-r43

**Published:** 2005-04-13

**Authors:** Ran Elkon, Sharon Rashi-Elkeles, Yaniv Lerenthal, Chaim Linhart, Tamar Tenne, Ninette Amariglio, Gideon Rechavi, Ron Shamir, Yosef Shiloh

**Affiliations:** 1 The David and Inez Myers Laboratory for Genetic Research, Department of Human Genetics, Sackler School of Medicine, Tel Aviv University, Tel Aviv, 69978, Israel; 2 School of Computer Science, The Chaim Sheba Medical Center and Sackler School of Medicine, Tel Aviv University, Tel Aviv, 69978, Israel; 3 Department of Pediatric Hemato-Oncology and Functional Genomics, The Chaim Sheba Medical Center and Sackler School of Medicine, Tel Aviv University, Tel Aviv, 69978, Israel

## Abstract

**Background:**

Gene-expression microarrays and RNA interferences (RNAi) are among the most prominent techniques in functional genomics. The combination of the two holds promise for systematic, large-scale dissection of transcriptional networks. Recent studies, however, raise the concern that nonspecific responses to small interfering RNAs (siRNAs) might obscure the consequences of silencing the gene of interest, throwing into question the ability of this experimental strategy to achieve precise network dissections.

**Results:**

We used microarrays and RNAi to dissect a transcriptional network induced by DNA damage in a human cellular system. We recorded expression profiles with and without exposure of the cells to a radiomimetic drug that induces DNA double-strand breaks (DSBs). Profiles were measured in control cells and in cells knocked-down for the Rel-A subunit of NFκB and for p53, two pivotal stress-induced transcription factors, and for the protein kinase ATM, the major transducer of the cellular responses to DSBs. We observed that NFκB and p53 mediated most of the damage-induced gene activation; that they controlled the activation of largely disjoint sets of genes; and that ATM was required for the activation of both pathways. Applying computational promoter analysis, we demonstrated that the dissection of the network into ATM/NFκB and ATM/p53-mediated arms was highly accurate.

**Conclusions:**

Our results demonstrate that the combined experimental strategy of expression arrays and RNAi is indeed a powerful method for the dissection of complex transcriptional networks, and that computational promoter analysis can provide a strong complementary means for assessing the accuracy of this dissection.

## Abstract 2

Microarray and RNAi technologies were applied to dissect a transcriptional network induced by DNA damage in human cells, revealing that two pivotal stress-induced transcription factors (NFκB and p53) mediated most of the damage-induced gene activation while a major transducer of the cellular responses to double strand breaks (ATM) was required for the activation of both pathways.

## Background

With completion of the sequencing of the human genome and those of many other organisms, research is shifting to functional genomics, that is, to gaining system-level understanding of the mechanisms by which gene products interact and regulate each other to produce coherent and coordinated physiological processes during normal development and in response to homeostatic challenges. Great progress has been made in the delineation of transcriptional regulatory networks [1-4], thanks to the maturation of gene-expression microarrays and the development of advanced computational approaches for analysis of the volumes of data generated by this technology. Another technological breakthrough that greatly enhances the ability to manipulate and characterize gene function in mammalian cells is the use of RNA interference (RNAi) for targeted silencing of specific genes [5-7]. The combination of global gene-expression profiling and RNAi-mediated silencing of key regulatory genes appears to offer a powerful tool for systematic dissection of transcriptional networks. However, recent studies pointed out that applying RNAi to mammalian cells triggers some nonspecific pathways [8-10] and affects an unpredicted number of off-targets [11] in addition to knocking-down the target of interest. This raises concern that nonspecific responses to small interfering RNAs (siRNA) might obscure the consequences of silencing the target of interest.

In this work, focusing on a DNA-damage-induced transcriptional network as a test case, we established human cells stably knocked-down for one of the major activators of the network, the protein kinase ATM (a gene that is mutated in the disease ataxia-telangiectasia), and for two key transcription factors that function downstream to it, NFκB and p53. Comparing gene-expression profiles measured in these cellular systems with and without exposure to a DNA damaging agent, we observed that NFκB and p53 mediated most of the damage-induced gene activation; that they controlled the activation of largely disjoint sets of genes; and that ATM was required for the activation of both pathways. Applying statistical tests coupled with computational promoter analysis, we demonstrated that the dissection of the damage-induced network into ATM/ NFκB - and ATM/p53-mediated arms was highly accurate. Thus, we show that this combined strategy is indeed a powerful method for the dissection of complex transcriptional networks.

## Results

**Figure 1:**
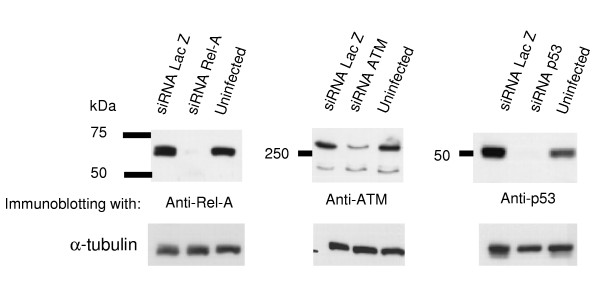
Western blotting analysis showing the reduction in protein levels encoded by mRNAs that were targeted by siRNAs. α-Tubulin was used as a loading control.

We established human cellular systems stably knocked-down for the ATM protein kinase, for the Rel-A subunit of NFκB, and for p53. Stable knock-down of the proteins was obtained by infecting HEK 293 cells with retroviral vectors expressing the corresponding short hairpin RNAs (shRNAs). Efficient reduction of protein levels was confirmed using western blotting analysis (Figure 1). Controls for our experiments were uninfected cells and cells infected with a vector carrying siRNA against *lacZ*, which has no significant homology to any human gene. Using Affymetrix Human Focus GeneChip arrays, we recorded gene-expression profiles in these cellular systems before and 4 hours after exposure to neocarzinostatin (NCS), an enediyne antitumor antibiotic that intercalates into the DNA and induces double-strand breaks (DSBs) [12]. Our dataset contains profile measurements for ten conditions: five cellular systems (two controls - uninfected cells and cells expressing siRNA against the bacterial enzyme LacZ - and cells knocked-down for Rel-A, p53 and ATM), each probed at two time points: without treatment and 4 hours after exposure to NCS. Each condition was measured in independent triplicates. Expression levels were computed using the Robust Multi-array Average (RMA) method [13] (see Materials and methods).

As a first step in our data analysis we searched for nonspecific responses to siRNA expression. We scanned the dataset for genes that were either consistently up- or downregulated in all four cells expressing siRNAs compared with their basal level in the uninfected control, all before exposure to NCS. We observed a subtle but statistically significant response to viral infection/siRNA expression. Very few genes were consistently responsive when a cutoff of 1.5-fold change was set, but lowering the threshold to 1.3-fold resulted in 20 consistently upregulated and 75 consistently downregulated genes in the infected cells (Additional data file 3). The threshold is low, but the number of genes that showed consistent response is significantly higher than expected by chance (in 1,000 datasets with randomly permutated entries for each gene, an average of 0.1 and 0.2 consistently up- and downregulated genes, respectively, were found). The set of consistently upregulated genes contained mainly genes involved in different aspects of cellular metabolism (Additional data file 2). The consistently downregulated genes included metabolic genes and genes that function in control of cell growth, signal transduction and stress responses (Additional data file 2). In contrast to some reports [8,10], we did not observe induction of the interferon pathway following the introduction of siRNA into the cells.

Next, we searched the dataset for genes that responded to the NCS treatment in the control uninfected cells and whose response was not disturbed by the introduction of siRNA into the cells: namely, genes that responded to the treatment in a coherent manner in the uninfected and the LacZ control cells. This damage-induced gene set (additional data file 4) contained 112 genes that were induced in both controls and met our criterion (see Materials and methods). Only seven genes met an analogous criterion for repression in response to NCS treatment; six of them are related to mitosis, presumably reflecting the activation of cell-cycle checkpoints in response to DNA damage (see Additional data file 4).

**Figure 2:**
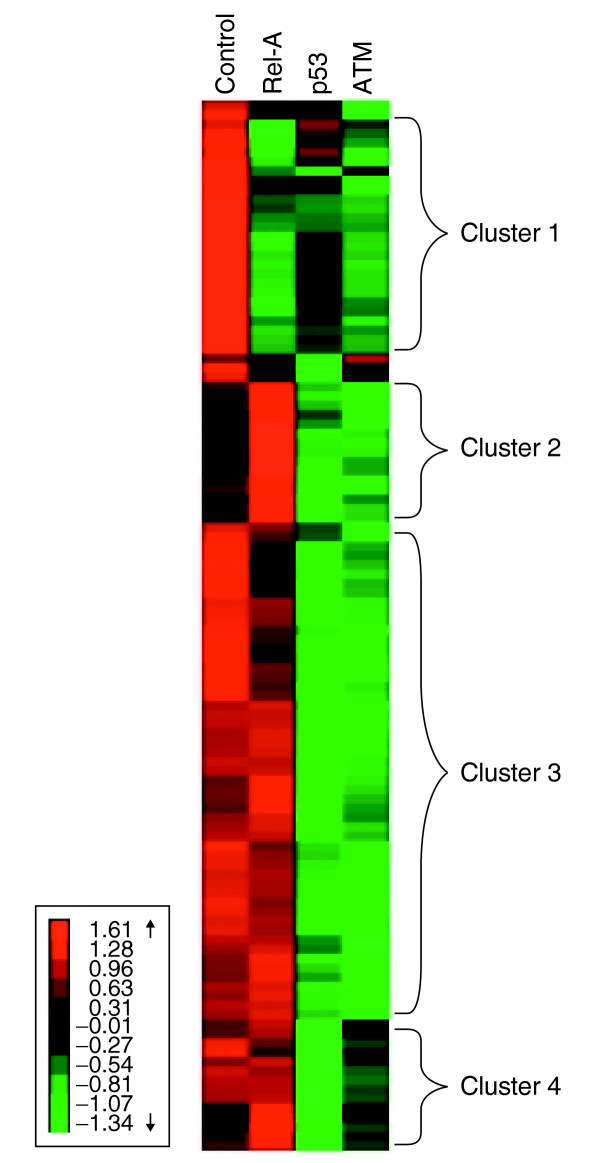
The four major expression patterns in the damage-induced gene set revealed by cluster analysis. For each of the 112 damage-induced genes, the fold change in expression level 4 h after NCS treatment was computed in uninfected cells and in the cells knocked-down for Rel-A, p53 and ATM, yielding a 112 × 4 data matrix, with the rows corresponding to genes. This matrix was subjected to hierarchical clustering after normalizing the rows to have mean = 0 and SD = 1. The heat map visually represents the normalized matrix after being clustered. Red, green and black entries represent above-, below- and near-average fold change of induction, respectively. Four prominent expression patterns are evident. Cluster 1 represents genes whose induction is strongly attenuated in cells knocked-down for Rel-A and ATM (compared to the response in the control uninfected cells), and only partially attenuated in cells knocked-down for p53. Cluster 2 represents genes whose response is attenuated in cells knocked-down for p53 and ATM, but augmented in cells knocked-down for Rel-A. Cluster 3 represents genes whose response is attenuated in cells knocked-down for p53 and ATM, but not affected by knocking-down Rel-A. Cluster 4 represents genes whose response is markedly attenuated in cells knocked-down for p53, and only partially attenuated in cells knocked-down for ATM.

We divided the expression level of each damage-induced gene at the 4-hour time point by its level in untreated cells in the same cellular system, and subjected the data to hierarchical cluster analysis. The damage-induced gene set was found to fall into four major response patterns (Figure 2): Cluster 1 contained 26 damage-induced genes whose response was strongly reduced in the absence of ATM and Rel-A, and only partially affected by the absence of p53. Cluster 2 contained 11 genes whose response was abolished in the absence of ATM and p53, but augmented in the absence of Rel-A, suggesting some negative regulatory effect for NFκB on their expression. Cluster 3 contained 46 genes whose response was markedly attenuated in the absence of ATM and p53, and not substantially affected by the absence of Rel-A. Cluster 4 contained 12 genes whose induction was strongly reduced in the absence of p53, partially affected by the absence of ATM, and not affected by the absence of Rel-A.

This analysis shows the following. First, the transcriptional network induced on exposure to NCS in these cells is almost completely mediated by NFκB and p53, and these two transcription factors induce nearly disjoint sets of genes: the former controls the induction of cluster 1 genes, the latter controls the induction of the genes in clusters 2-4. Second, ATM is required for the activation of a major part of the damage-induced transcriptional program, comprising both the NFκB and p53 response arms (the activation of clusters 1-3 genes is ATM-dependent). Third, there is some cross-talk between the NFκB and p53 pathways: the absence of p53 partially reduces the induction of the NFκB arm (cluster 1), suggesting a positive effect of p53 on the induction of the NFκB mediated response; and the absence of Rel-A leads to increased activation of a subset of the p53-mediated arm (cluster 2), pointing to a negative regulatory role for NFκB in the induction of these genes.

The cluster analysis identified transcriptional responses mediated by both ATM/NFκB and ATM/p53. We sought to demonstrate that this dissection of the ATM-mediated transcriptional network induced by DNA damage is precise and cannot reasonably be ascribed to some nonspecific or off-target effects. To this end, we examined the effect of knocking-down Rel-A and p53 on several of their respective known direct targets that were included in the damage-induced genes set. Table 1a shows that knocking-down Rel-A and ATM significantly blocked the induction of known NFκB target genes, whereas knocking-down p53 had a much milder effect on their induction. Table 1b shows that knocking-down p53 and ATM specifically blocked the induction of known p53 target genes, whereas knocking-down Rel-A did not disrupt their induction (and even augmented it for some genes). Results of quantitative real-time reverse transcription PCR (RT-PCR), performed to validate the microarray results for these genes, were in good agreement with the microarray data in most cases; the magnitudes of induction differed between the two experimental systems, but the dependency of transcriptional induction on the various regulators was similar for 10 out of 13 genes examined.

To confirm the accuracy of the network dissection obtained by our experimental setup, we applied the PRIMA tool to our dataset. PRIMA, a computational promoter analysis tool recently developed by us [14], identifies transcription factors whose binding-site signatures are significantly more prevalent in a given set of promoters than expected by chance (see Materials and methods). In particular, promoters of genes assigned to cluster 1, which represents an ATM/NFκB-dependent response, were specifically and highly significantly enriched for the binding site signature of NFκB (Table 2), whereas p53-dependent clusters 3 and 4 were specifically enriched for the binding site of ATF2. ATF2 regulates transcription after heterodimerization with either ATF3 or c-Jun [15].

Notably, in our dataset the induction of both *ATF3 *and *c-Jun *was p53-dependent (Table 1b); hence the enrichment for this signature probably reflects a second wave of transcriptional regulation controlled by these transcription factors, whose induction is mediated by p53. This agrees with other studies that reported a p53-dependent activation of ATF3 in response to DNA damage [16,17]. PRIMA did not identify enrichment for the p53-binding-site signature in the p53-dependent clusters. It is possible that PRIMA is not sensitive enough to detect p53 enrichments because of the complex nature of the binding sites for p53 [18] or their relatively long distance from the transcription start sites (many experimentally validated p53-binding sites are located outside the promoter region included in PRIMA analysis). However, using the same parameters, PRIMA did identify significant enrichment for p53-binding signature in several other microarray datasets that we analyzed (data not shown). We therefore believe that p53 signature is not over-represented in these clusters, suggesting that p53 in the cells we used exerts its direct effect on a limited number of target genes, which are then further expanded into a wider network of transcriptional responses mediated mainly by ATF/Jun.

## Discussion

The fine dissection of complex transcriptional responses has been a long-standing challenge in the signal transduction field. External and internal stimuli may activate complex networks whose analysis by traditional biochemistry can be daunting. High-throughput methods developed for functional genomics combined with powerful computational tools hold promise for deciphering such networks. The DNA damage response is an appropriate target for such an analysis. This highly branched signaling network spans numerous aspects of cellular metabolism and involves a vigorous wave of gene transcription across the genome.

In this study we have demonstrated the combined use of RNAi and microarray technologies and a recently developed computational tool to dissect the ATM-dependent transcriptional response following the induction of DSBs in DNA. RNAi technology has recently revolutionized biological research, but questions have been raised about the specificity of RNAi-mediated gene repression [8-11]. One way to filter out off-target effects is to use several different siRNA sequences against the same target on the assumption that completely different siRNAs will not induce the same off-target effects [7,11]. Following this logic, dissection of a signaling pathway that is mediated by several regulators using independent targeting of these regulators should similarly boost confidence. In this case, overlapping sets of genes whose expression is attenuated by knocking down different regulators are unlikely to be a result of off-target effects. It is also important to show that the observed effects are not a general consequence of the expression of siRNAs in the cells.

Our general goal is to dissect the DNA damage-induced transcriptional response in various cell types and tissues. In this study we focused on two arms of the this network whose induction is specifically mediated by the ATM/NFκB and the ATM/p53 regulators. First, we identified a set of genes whose induction in response to DNA damage was abrogated in cells knocked-down for two different components of the damage-induced signaling pathway, ATM and the Rel-A subunit of NFκB. Importantly, the induction of these genes was not disrupted in cells expressing siRNA against LacZ and was only mildly attenuated in cells knocked-down for p53, indicating that the loss of induction was not a general nonspecific consequence of siRNA expression. Moreover, computational promoter analysis showed that the set of promoters of these genes was highly and specifically enriched for the binding site signature of NFκB, providing independent evidence of the accuracy of this analysis. We then identified a set of genes whose induction in response to DNA damage was significantly abrogated in cells knocked-down for ATM and p53, but not in cells knocked-down for the Rel-A subunit of NFκB, or in the LacZ control. Again, it is unlikely this dissection of the ATM/p53-mediated arm can be ascribed to nonspecific or off-targets effects. According to computational promoter analysis, this set was highly enriched for the binding signature of ATF2/ATF3/Jun, a secondary transcriptional pathway whose induction was indeed p53-dependent in our data. This observation is in agreement with several studies reporting p53-dependent activation of this transcriptional pathway in response to DNA damage [16,17]. However, evidence suggests that p53-dependence of the induction of the ATF2/ATF3/Jun pathway depends on the cellular context, the type of DNA lesion, or the extent of damage, as p53-independent induction of this pathway was observed in other studies [19,20].

Evidence suggests that the sets of genes regulated by specific transcription factors depend on cell type and tissue context (see [21,22]). We are currently extending the analysis to various types of cell lines treated with a variety of DNA-damaging agents. Initial results indicate a marked cell-type specificity of the transcriptional response to DNA damage. The strategy presented here holds promise for disclosing and better understanding of this specificity.

## Conclusions

Our analysis demonstrates that the combination of RNAi-targeting of key regulators, gene-expression profiling using microarrays, and computational promoter analysis is an informative method for the dissection of transcriptional networks in mammalian cellular systems despite the potential nonspecific and off-target effects of the RNAi technology. Targeting the primary activator of a DNA damage response network, the ATM protein kinase, and two key transcription factors that function downstream to it, p53 and NFκB, we showed that while the upstream regulator was indeed required for the induction of much of the network, the two downstream regulators mediated the activation of largely disjoint sets of genes. Thus, we dissected the network into two major arms. Statistical tests coupled with computational promoter analysis showed that this dissection was highly accurate.

## Materials and methods

### Establishment of siRNA knocked-down cellular systems

The following DNA fragments expressing shRNAs were cloned in the pSUPER retroviral vector [23,24], specifically designed to express siRNAs:

ATM_I (7218) 5'-GATCCCCCTGGTTAGCAGAAACGTGCTTCAAGAGAGCA CGTTTCTGCTAACCAGTTTTTGGAAA-'3.

ATM_II (p480): 5'-GATCCCCGATACCAGATCCTTGGAGATTCAAGAG ATCTCCAAGGATCTGGTATCTTTTTGGAAA-3', a generous gift from R. Agami. (ATM level was knocked-down using a combination of two different siRNAs.)

Rel_A: 5'-GATCCCCGAAGAGTCCTTTCAGCGGATTCAAGAGATCCGCTGAAAG GACTCTTCTTTTTGGAAA -3'.

p53: 5'-GATCCCCGACTCCAGTGGTAATCTACTTCAAGAGAGTAGATTACCACTG GAGTCTTTTTGGAAA-'3 (previously described in Brummelkamp *et al. *[24]).

LacZ: 5'-GATCCCCAAGGCCAGACGCGAATTATTTCAAGAGAATAATTCGCGTCT GGCCTTTTTTTGGAAA-3'.

HEK293 cells were transfected with ecotropic receptor expressing vector, infected with packaged viral particles, and selected with puromycin or hygromycin. Once stabilized, the cells were grown without selection.

### Sample preparation and microarray hybridization

Cells were treated for 4 h with 200 ng/ml of NCS. Total RNA was isolated using TRIzol reagent (Life Technologies) and treated with DNase I (DNA free, Ambion). RNA was then purified using PLG tubes (Eppendorf), phenol/chloroform extracted, ethanol-precipitated and quantitated. The integrity of the RNA and the absence of contaminating genomic DNA were examined using gel electrophoresis. Expression profiles were recorded using Affymetrix Human Focus GeneChip arrays, which represent some 8,500 well annotated genes. Targets for hybridization to the microarrays were prepared using standard methods according to the manufacturer's instructions. Hybridization and scanning were performed as recommended by the manufacturer. All samples were probed in independent triplicates.

### Computation of gene expression levels from microarray signals

Expression levels were computed using the RMA method [13] that was run from the BioConductor package [25]. The dataset was submitted to the Gene Expression Omnibus database [26] with accession number GSE1676. We preferred to use RMA over Affymetrix' MAS5 for two reasons. First, several studies have indicated that the mismatch signals are correlated with the mRNA concentration of their corresponding gene; that is, they themselves contain information on the expression level of the genes. Hence, subtracting their signals from the perfect-match ones, as MAS5 does, may add noise to the measurement and therefore be counterproductive [13]. RMA ignores the mismatch probes and computes expression levels based only on perfect match signals. When we examined the mismatch probe signals for several genes activated by the NCS treatment, we found that these signals indeed increased, in a manner correlated with the increase exhibited by their corresponding perfect-match signals (Additional data file 1). Second, whereas MAS5 uses global scaling to normalize between arrays, RMA applies the quantile normalization that was demonstrated to perform better [27]. Comparison of expression levels computed by MAS5 and RMA showed that RMA reduced noise between replicates (Additional data file 1), as well as the range of fold-changes in gene expression after the treatment (Additional data file 2).

Probe sets that received 'Absent' calls in all chips were filtered out, leaving 6,002 probe sets for subsequent steps of the data analysis. Averaging expression levels over replicates, our dataset contained measurements for ten conditions: five cellular systems (uninfected and the LacZ control cells and cells knocked-down for Rel-A, p53 and ATM), each probed at two time points: without treatment and 4 h after exposure to NCS.

### Definition of the damage-responding gene set

We defined the damage-responding gene set as all genes whose expression levels changed by at least 1.5-fold in one control (either the uninfected or the LacZ-infected cells), and at least 1.4-fold in the same direction in the other control. A total of 112 genes that were induced in both controls met this criterion and are referred to as the damage-induced gene set (Additional data file 4). Only seven genes met an analogous criterion for repression in response to NCS treatment (Additional data file 4). We chose thresholds of 1.5 and 1.4 - lower than those usually used in microarray analysis - because the RMA method significantly narrows the distribution of expression levels and of the fold changes compared to Affymetrix' MAS5 package (Additional data files 1 and 2). Although the thresholds are low, the expected false-positive rate in our damage-induced gene set is low: not a single gene passed this criterion when it was applied to expression levels measured 30 min after exposure of the cells to NCS (data not shown). In addition, this number is significantly higher than expected at random: in 1,000 datasets with randomly permuted entries for each gene, the average number of genes that met this criterion was 14.1.

### Cluster analysis

For each of the 112 damage-induced genes, induction fold-change of expression level after NCS treatment was computed in the control uninfected cells and in the cells knocked-down for Rel-A, p53 and ATM. The expression level of each damage-induced gene at the 4-h time point was divided by its level at the 0 time point in the same cellular system, yielding a 112 × 4 data matrix, with rows corresponding to genes. We normalized each row to mean = 0 and standarad deviation (SD) = 1, and subjected the normalized matrix to average-linkage hierarchical clustering using the EXPANDER package for microarray data analysis [28,29].

### GO functional gene annotations

The gene ontology (GO) annotations of the genes were extracted using the DAVID utility [30].

### Computational promoter analysis

Computational promoter analysis was done using PRIMA software, described in detail in Elkon *et al. *[14] and available at [31]. In brief, given target and background sets of promoters, PRIMA performs statistical tests aimed at identifying transcription factors whose binding sites are significantly more abundant in the target set than in the background set. PRIMA uses position weight matrices (PWMs) as models for regulatory sites that are bound by transcription factors. PWMs that represent human or mouse transcription-factor-binding sites were obtained from the TRANSFAC database [32]. The four gene clusters were used as target sets, and the entire collection of genes present on the chip (after filtering out those that got Absent calls in all chips) served as the background set in PRIMA tests. Putative promoter sequences corresponding to all known human genes were extracted from the human genome (Ensembl, version 19, Feb 2004), using a Perl script based on the application programming interface provided by the Ensembl project [33]. PRIMA tests were confined to 800 bp upstream to the putative genes' transcription start sites. Repetitive elements were masked out. Both strands were scanned.

### Quantitative real-time RT-PCR

Five micrograms of total RNA were used for cDNA synthesis by oligo(dT) and SuperScript II RNase H^- ^reverse transcriptase (Life Technologies). Quantitative real-time PCR using SYBR Green PCR master mix (Applied Biosystems) was performed with ABI PRISM 7900HT sequence detection system (Applied Biosystems). The comparative *C*_t _method was used for quantification of transcripts according to the manufacturer's protocol. Measurement of Δ*C*_t _was performed in triplicate. We used glyceraldehyde-3-phosphate dehydrogenase (GAPDH) as the control gene for normalization. Primer pairs used in this study are given in Additional data file 2.

## Additional data files

The following additional data are available with the online version of this paper. Additional data file 1 contains two figures showing the microarray results and their analysis. Additional data file 2 contains tables showing GO categories of affected genes, comparison between MAS5 and RMA computation of expression levels, primers used for real-time RT-PCR and the sequences of the shRNAs use in this study. Additional data file 3 contains a table listing genes whose expression was affected by infection of the cells with the shRNA-expressing retroviral vectors. Additional data file 4 contains a table listing the genes induced in both controls in in response to NCS treatment, and their assignment into the four clusters.

## Supplementary Material

Additional File 1 Two figures showing the microarray results and their analysis. Supplementary Figure 1. Perfect-match (PM) and mismatch (MM) probe signals measured prior to and 4 hours after treatment with NCS. These signals are shown for four genes that were induced by the NCS treatment. As can be seen, mismatch signals were increased as well, pointing that they too contain information on gene expression level. Supplementary Figure 2. Comparison between RMA and MAS 5 computed signals. M vs. A plots (as introduced by Speed's lab ) based on expression levels that were computed by MAS5 or RMA for comparison between: (i) two replicated chips (C0a vs. C0b) (ii) post-treatment vs. pre-treatment chips (C0a vs. C4a), and (iii) same as (ii) but expression levels were averaged on triplicate chips at both time points. In all comparisons, the fold induction distributions (represented by the Y-axis) were markedly narrower when expression levels were computed by RMA. Distributions based on MAS5 were especially noisy in the low intensity genes. Click here for file

Additional File 2 Tables showing GO categories of affected genes, comparison between MAS5 and RMA computation of expression levels, primers used for real-time RT-PCR and the sequences of the shRNAs use in this study. Supplementary Table B. GO categories of the genes that were upregulated in response to infection of the cells with shRNA-expressing retroviral vectors. Supplementary Table C. GO categories of the genes that were downregulated in response to infection of the cells with the shRNA-expressing retroviral vectors. Supplementary Table E. Comparison between MAS 5 and RMA computation of expression levels. Supplementary Table F. Primers used for quantitative real-time RT-PCR assays. Supplementary Table G. Sequences of shRNAs used in this study. Click here for file

Additional File 3 A table listing genes whose expression was affected by infection of the cells with the shRNA-expressing retroviral vectors. Supplementary Table A. Genes whose expression was affected by infection of the cells with the shRNA-expressing retroviral vectors. Click here for file

Additional File 4 A table listing the genes induced in both controls in in response to NCS treatment, and their assignment into the four clusters. Supplementary Table D. List of the 112 genes that were induced in both controls in response to NCS treatment, and their assignment into the four clusters. Click here for file

## Figures and Tables

**Table 1:** Fold change in gene expression after 4 h exposure to NCS as measured by microarrays and by quantitative real-time RT-PCR

Gene	Affy_ID	Fold induction microarray					Fold induction RT-PCR			
			
		C	LacZ	Rel-A (NFκB)	p53	ATM	C	Rel-A (NFκB)	p53	ATM
**(a) **Known direct targets of NFκB										
TNFAIP3	202644_s_at	8.28	5.34	1.15	3.02	1.19	9.5	1.1	9.5	0.9
RELB	205205_at	3.7	2.89	0.82	2.95	0.91	15.7	6.0	21.3	2.5
TNFRSF9	207536_s_at	4.01	3.5	1.1	2.08	1.21	14.3	3.5	11.0	1.4
NFKBIA	201502_s_at	4.61	5.4	1.26	2.67	1.02	4.2	1.7	4.5	1.2
CD83	204440_at	3.46	2.99	1.0	1.73	1.06	6.5	1.0	5.7	1.3
IER3	201631_s_at	4.44	5.12	1.43	2.35	1.44	6.6	1.8	3.4	1.8
										
**(b) **Known direct targets of p53										
ATF3	202672_s_at	3.44	3.74	7.03	1.54	1.47	5.2	5.9	1.6	1.6
EGR1	201694_s_at	2.78	1.77	6.77	1.04	1.02	4.4	13.4	0.7	2.4
JUN*	213281_at	2.01	1.45	2.71	1.36	1.25	6.6	3.9	0.64	2.5
FOS	209189_at	1.72	1.42	2.22	1.07	1.22	3.4	13.1	3.4	1.9
ETR101*	202081_at	1.97	2	2.6	1.06	1.13	2.0	3.0	1.4	1.4
GADD45A	203725_at	2.36	2.07	2.00	1.07	1.22	1.8	2.3	1.8	1.3
DUSP1	201041_s_at	2.06	2.57	3.45	1.11	1.22	2.2	4.5	2.0	1.9

*These genes are not reported as direct targets of p53 but are known to be functionally related to p53.

**Table 2:** Significantly enriched transcription factor binding site signatures in promoters of co-clustered genes

Cluster	Number of genes*	Dependence of gene induction^†^			Binding-site enrichment^‡^	
			
		ATM	Rel-A (NFκB)	p53	NFκB (M00054)	ATF2 (M00179)
1	26	++	++	+	9.7 (6.0 × 10^-12^)	-
3	46	++	-	++	-	2.9 (2.7 × 10^-5^)
4	12	+	-	++	-	6.6 (3.6 × 10^-6^)

*Number of genes with promoter sequence data. ^†^Strong attenuation in induction of the cluster's genes in the respective cells is denoted by ++; partial attenuation is denoted by +; and no attenuation by -. ^‡^The ratio between transcription-factor hit prevalence in the cluster and in the background sets of promoters, and its *p*-value (accession numbers for transcription-factor binding site models are from TRANSFAC DB).
