# Supplementary figures and images for: Dissection of a DNA-damage-induced transcriptional network using a combination of microarrays, RNA interference and computational promoter analysis
Source: Genome Biol. 2005 Apr 13;6(5):R43. doi: 10.1186/gb-2005-6-5-r43 (PMC1175955; doi:10.1186/gb-2005-6-5-r43)

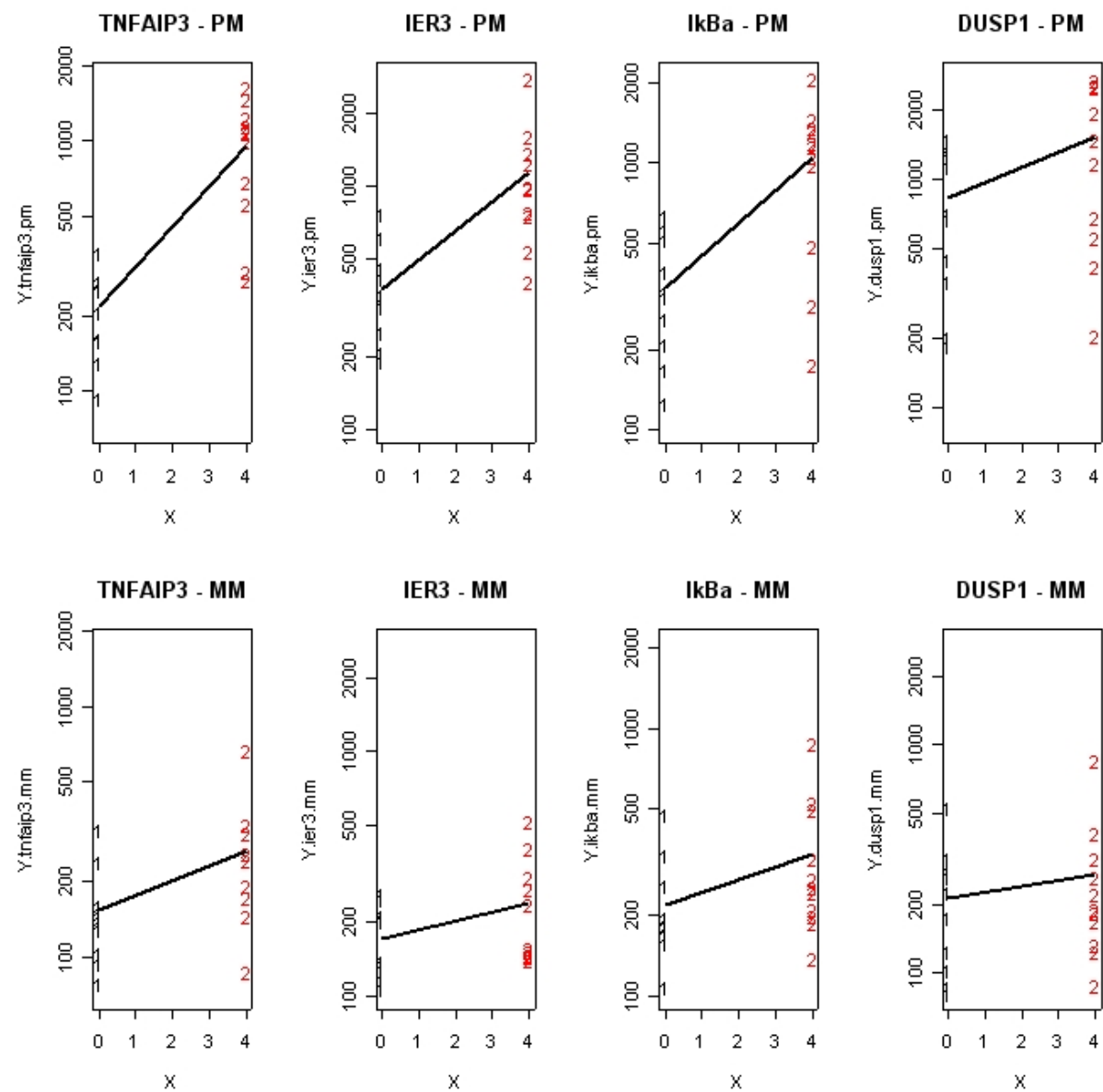

Supplementary Fig 1. Elkon et al.

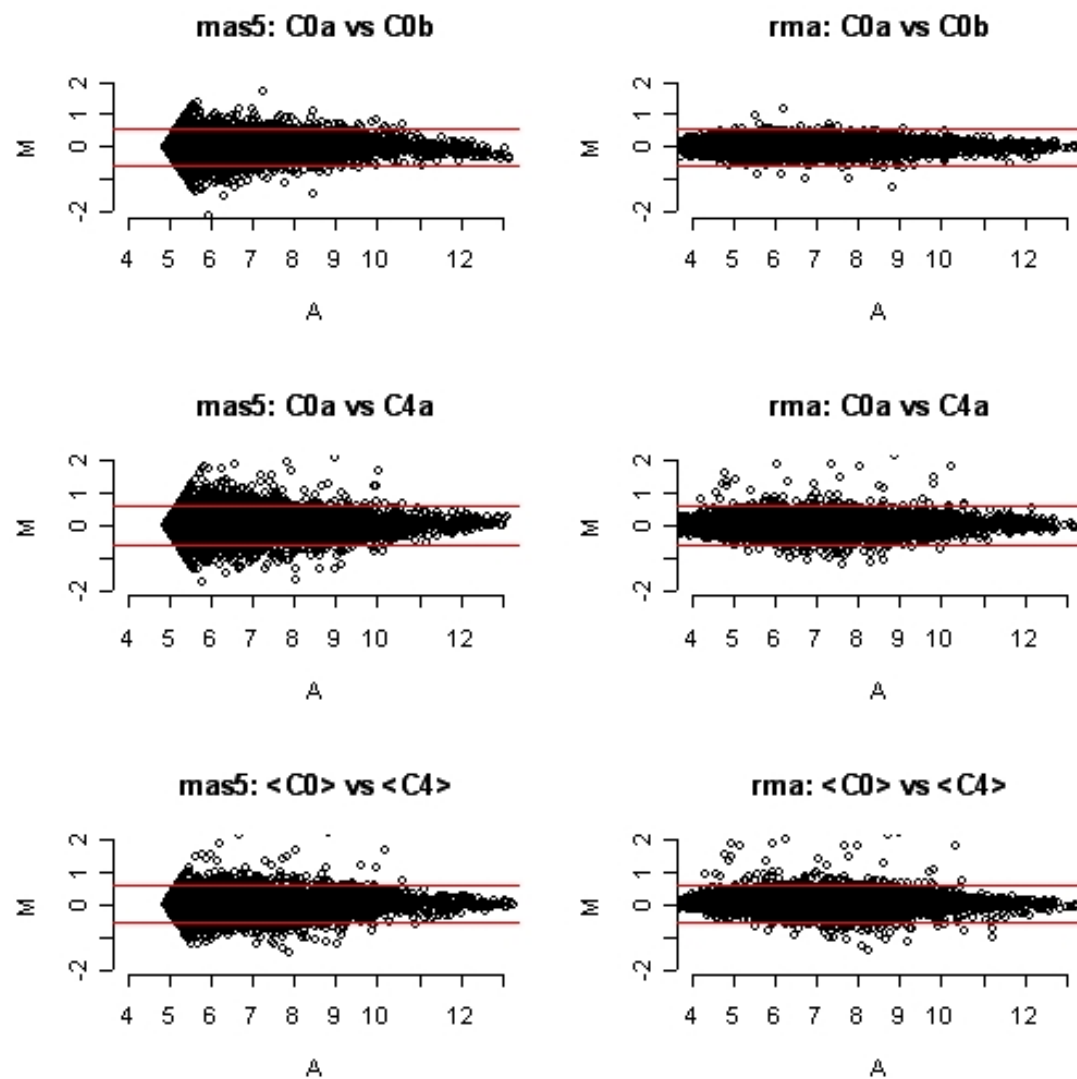

Supplementary Fig 2. Elkon et al.

Supplement: Additional File 1 — Two figures showing the microarray results and their analysis. Supplementary Figure 1. Perfect-match (PM) and mismatch (MM) probe signals measured prior to and 4 hours after treatment with NCS. These signals are shown for four genes that were induced by the NCS treatment. As can be seen, mismatch signals were increased as well, pointing that they too contain information on gene expression level. Supplementary Figure 2. Comparison between RMA and MAS 5 computed signals. M vs. A plots (as introduced by Speed's lab ) based on expression levels that were computed by MAS5 or RMA for comparison between: (i) two replicated chips (C0a vs. C0b) (ii) post-treatment vs. pre-treatment chips (C0a vs. C4a), and (iii) same as (ii) but expression levels were averaged on triplicate chips at both time points. In all comparisons, the fold induction distributions (represented by the Y-axis) were markedly narrower when expression levels were computed by RMA. Distributions based on MAS5 were especially noisy in the low intensity genes. [file gb-2005-6-5-r43-S1.pdf]
