# Supplementary material for: Dissection of a DNA-damage-induced transcriptional network using a combination of microarrays, RNA interference and computational promoter analysis
Source: Genome Biol. 2005 Apr 13;6(5):R43. doi: 10.1186/gb-2005-6-5-r43 (PMC1175955; doi:10.1186/gb-2005-6-5-r43)
Supplement: Additional File 2 — Tables showing GO categories of affected genes, comparison between MAS5 and RMA computation of expression levels, primers used for real-time RT-PCR and the sequences of the shRNAs use in this study. Supplementary Table B. GO categories of the genes that were upregulated in response to infection of the cells with shRNA-expressing retroviral vectors. Supplementary Table C. GO categories of the genes that were downregulated in response to infection of the cells with the shRNA-expressing retroviral vectors. Supplementary Table E. Comparison between MAS 5 and RMA computation of expression levels. Supplementary Table F. Primers used for quantitative real-time RT-PCR assays. Supplementary Table G. Sequences of shRNAs used in this study. [file gb-2005-6-5-r43-S2.pdf]

Supplementary Table B. GO categories of genes that were up-regulated in all infected cells compared to their basal level in the uninfected control

GO CATEGORY	NUMBER
protein metabolism	6
nucleobase, nucleoside, nucleotide and nucleic acid metabolism	5
catabolism	4
biosynthesis	3
cell growth and/or maintenance	3
lipid metabolism	3
unclassified	5

Supplementary Table C. GO categories of genes that were down-regulated in all infected cells compared to their basal level in the uninfected control

**GO CATEGORY**	**NUMBER**
cell growth and/or maintenance	19
nucleobase, nucleoside, nucleotide and nucleic acid metabolism	17
response to external stimulus	13
cell adhesion	10
signal transduction	10
protein metabolism	9
immune response	8
organogenesis	6
regulation of cell proliferation	6
response to stress	6
catabolism	4
cell death	4
regulation of cell growth	4
cell motility	3
cell-cell signaling	3
neurophysiological process	3
organismal movement	3
phosphorus metabolism	3
response to endogenous stimulus	3
unclassified	23

Supplementary Table E. MAS5 vs RMA

		(I) C_0a vs C_0b	(II) C_0a vs C_4a	(III) <C_0> vs <C_4>
**MAS5**	**Up**	278	342	148
	**Down**	202	318	251
**RMA**	**Up**	11	74	95
	**Down**	7	58	68

Comparison of expression levels computed by MAS5 and RMA. Number of genes whose expression level was increased (Up) or decreased (Down) 1.5-fold (I) when two replicates of control cells are compared at time 0, (II) in a single chip prior to and 4 hrs after NCS treatment, and (III) in triplicate averaged chips of samples prior to and after NCS treatment. Expression levels below 30 were set to 30. Note the 25-fold decrease in the noise between two control replicates measured by the two methods (see also Supplementary Fig 3).

Supplementary Table F. Primers used in quantitative real-time RT-PCR assays.

Gene name	Locuslink ID	Forward primer (5'→3')	Reverse primer (5'→3')
CD83 (450-602)	9308	GTCTCCTGGGTCAAGTTATTGGA	AGTGTTTCGGATCTTCAGGGAATAG
IER3 (211-339)	8870	GCAGCCGCAGGGTTCTC	CCTCTTCAGCCATCAGGATCTG
NFκBIa (389-469)	4792	GGAGACCTGGCTTTCCTCAACT	TTCTGGCTGGTTGGTGATCAC
RelB (1299-1349)	5971	GCCATTGCCTTTCACGTACCT	TCCACGCCGTAGCTGTCAT
TNFAIP3 (2071-2181)	7128	GGAAGCACCATGTTTGAAGGATAC	TCTGCGCTGGCTCGATCT
TNFRSF9 (659-759)	3604	TGCGAGAGAGCCAGGACACT	AGAAACGGAGCGTGAGGAAG
ATF3 (462-542)	467	GCTGCAAAGTGCCGAAACA	AGCATTCACACTTTCCAGCTTCT
DUSP1 (959-1129)	1843	CAACGAGGCCATTGACTTCATAG	ATGCTTCGCCTCTGCTTCA
EGR1 (1459-1531)	1958	GCCTGCGACATCTGTGGAA	GCCGCAAGTGGATCTTGGTA
ETR101 (1778-1890)	9592	CCCTCAGACACACGGACACA	AGCAACTGGTCTCAGCTCAGC
Fos (533-593)	2353	GGGCAAGGTGGAACAGTTATCT	CCTTTCCCTTCGGATTCTCCT
GADD45A (669-803)	1647	TGCTGGTGACGAATCCACA	TCAGATGCCATCACCGTTCA
cJun (1270-1320)	3725	TGGAGCGCCTGATAATCCA	TCGGCGTGGTGGTGATG
GAPDH (943-1043)	2597	ACCCACTCCTCCACCTTTGA	CTGTTGCTGTAGCCAAATTCGT

Primers were designed with the PRIMER EXPRESS^{TM} 1.0 software (PE Applied Biosystems, Foster city, CA, USA).

Supplementary Table G. Sequences of shRNAs used in this study.

ATM_I (7218) 5'-gatccccctggttagcagaaacgtgcttcaagagagcacgtttctgctaaccagtttttggaaa–'3.

ATM_II (p480): 5'-gatccccgataccagatccttggagattcaagagatctccaaggatctggtatctttttggaaa-3'.

(ATM level was knocked-down using a combination of two different siRNAs).

Rel_A: 5'-gatccccgaagagtcctttcagcggattcaagagatccgctgaaaggactcttctttttggaaa -3'

p53: 5'-gatccccgactccagtggtaatctacttcaagagagtagattaccactggagtctttttggaaa–'3 (previously described in Brummelkamp et al.(Brummelkamp et al., 2002)).

LacZ: 5'-gatccccaaggccagacgcgaattatttcaagagaataattcgcgtctggcctttttttggaaa-3'

Brummelkamp, T.R., Bernards, R. and Agami, R. (2002) Stable suppression of tumorigenicity by virus-mediated RNA interference. *Cancer Cell*, **2**, 243-247.
